# Validation of a machine-learning-based algorithm to predict preeclampsia-related adverse outcomes on a real-world dataset

**DOI:** 10.1007/s00404-025-08261-7

**Published:** 2026-02-06

**Authors:** Ameli Hoyler, Oliver Rieger, Max Hackelöer, Mark Neznansky, Wolfgang Henrich, Lisa Lorenz-Meyer, Stefan Verlohren

**Affiliations:** 1 Department of Obstetrics, Charité - Universitätsmedizin Berlin, Berlin, Germany; 2 Department of Obstetrics and Fetal Medicine, University Medical Center Hamburg-Eppendorf, Hamburg, Germany

**Keywords:** Machine learning, Artificial intelligence, sFlt-1/PlGF-ratio, Preeclampsia, Algorithms

## Abstract

**Purpose:**

Preeclampsia is a major obstetric disorder. Machine learning (ML) models incorporating angiogenic biomarkers show promise in predicting related adverse outcomes, but refinement is needed for clinical use. This study aimed to reduce features to a clinically meaningful set and to develop and validate predictive endpoints for preeclampsia-associated outcomes.

**Methods:**

A model with a reduced feature set was derived from a training cohort of 1,634 patients (2,

412 visits) and then tested on a validation cohort of 402 patients (540 visits). Three machine learning models were developed to predict (1) adverse outcomes overall, (2) delivery within 14 days before 34 weeks of gestation, and (3) delivery within 7 days after 34 weeks, using 13 features versus 114 originally.

**Results:**

Reduced-feature models demonstrated comparable accuracy to original models across all endpoints. Model 1 (any adverse outcome) achieved an Area Under the Receiver Operating Characteristic Curve (AUROC) of 0.92 (95% CI: 0.88–0.96) in training and 0.89 (95% CI: 0.84–0.93, *p* = 0.31) in the validation cohort, respectively. For delivery within 14 days, the AUROC was 0.92 (95% CI: 0.87–0.96) in training and 0.85 (95% CI: 0.78–0.92) in validation (*p* = 0.13). Delivery within 7 days showed AUROCs of 0.79 (95% CI: 0.70–0.87) and 0.80 (95% CI: 0.75–0.85) (*p* = 0.78).

**Conclusion:**

A machine learning model with a significantly reduced number of features can accurately predict clinically relevant preeclampsia outcomes. The identified endpoints (timing of delivery and adverse events) could support clinical decision-making and help reduce maternal and neonatal morbidity and mortality.

## What does this study add to the clinical work

**Table:** 

An ML-model using only 13 routinely available features can accurately predict preeclampsia-related AOs and timing of delivery, performing comparably to more complex models. This approach has potential for integration into clinical care to support timely interventions and improve maternal and neonatal outcomes. However, further prospective evaluation is warranted to assess safety, utility and cost-effectiveness.	

## Introduction

Preeclampsia affects 2–8% of pregnancies worldwide [1, 2] and is a leading cause of maternal and neonatal morbidity and mortality [3], resulting in 76,000 maternal and 500,000 neonatal deaths annually [1], many of which are preventable. A study from the Netherlands showed substandard care contributing to up to 96% of maternal deaths from preeclampsia [4].

Preeclampsia is a multisystem pregnancy disorder with variable presentation, ranging from mild disease to fatal complications such as eclampsia. It is typically diagnosed as new-onset hypertension after 20 weeks gestation with proteinuria, or other organ dysfunction [5]. Treatment consists of anti-hypertensive medication and magnesium to prevent eclampsia. While delivery remains the only definitive intervention to halt the progression of preeclampsia, it does not constitute a cure, as symptoms and complications may persist or develop after delivery. Timing delivery requires balancing maternal risk against neonatal prematurity [6]. Patients with preeclampsia are often closely monitored either at home or in a hospital setting to detect disease progression as early as possible, yet accurate identification of high-risk patients remains an unmet need, resulting in insufficient medical care for patients and high economic costs [7–9]. Angiogenic factors such as the sFlt-1/PlGF-ratio improve the prediction of preeclampsia and related adverse outcomes (AO) [10]. According to the PROGNOSIS study, a ratio of ≤ 38 is associated with a negative predictive value of 99.3% for the development of preeclampsia within the following week [11].

Given the heterogeneity of symptoms and clinical presentation, multi-marker models have proven to be successful in predicting preeclampsia [12]. Machine Learning (ML) is a novel approach and has been employed by us [13] and others [14–16]. Schmidt. et al. created an ML model using the gradient-boosted tree method (GBTree) to predict a composite of fetal or maternal AOs, achieving an AUROC 0.82 ± 0.03, outperforming the current standard of care [13]. However, their model used 114 features, limiting clinical applicability. We therefore examined whether a reduced-feature model could maintain predictive accuracy. Additionally, we built two reduced-feature models to predict time to delivery.

## Material and methods

### Study cohorts

We retrospectively recruited two cohorts from the electronic health records of the Department of Obstetrics, Charité—Universitätsmedizin Berlin. The training cohort was adapted from Dröge et al. [17], which also served as the basis for the algorithm created by Schmidt et al.[13]. Patients were recruited from July 2010 to June 2019 and will be referred to as “training cohort”. We excluded all visits with more than 50% missing values. The second cohort was recruited from July 2019 to October 2020 and will be referred to as “validation cohort”. Both cohorts represent pregnant women from the Berlin-Brandenburg region who presented to the hospital with symptoms or clinical suspicion of preeclampsia. The same Inclusion and Exclusion Criteria were applied. The study was approved by the Ethics Committee of Charité – Universitätsmedizin Berlin (approval number EA2/064/20).

*Inclusion and exclusion criteria*

We included pregnant women with singleton pregnancy and a gestational age of ≥ 20 weeks, who were 18 years or older, and presented with symptoms of or suspected preeclampsia. Included in our analysis were all cases for whom the pregnancy outcome was recorded and measurements of the sFlt-1/PlGF-ratio were available. Suspected preeclampsia was defined as elevated blood pressure (systolic ≥ 140 mmHg, diastolic ≥ 90 mmHg)or preexisting hypertension without further organ manifestation of preeclampsia. In the absence of hypertension, preeclampsia was suspected if one of the following findings occured: proteinuria detected by urine dipstick of “2 + ” or ≥ 300 mg/l in 24-h urine sampling or a protein/creatinine ratio of more than 300 mg/g, laboratory results suspicious of preeclampsia (elevated liver enzymes, low platelets), symptoms of preeclampsia such as headache, visual disturbances, upper abdominal pain, edema or weight gain, fetal abnormalities such as fetal growth restriction (< 10 percentile, FGR) or being diagnosed as small for gestational age (SGA). FGR was defined as an estimated fetal weight below the 10th percentile and/or a flattening of the growth curve in combination with oligohydramnios and/or pathological Doppler findings of the umbilical or uterine arteries, while SGA was defined as an estimated fetal weight at or below the 10th percentile without additional pathological findings. Pregnancies with known fetal anomalies or chromosomal disorders were excluded, as well as patients with a known clinical diagnosis of preeclampsia necessitating a delivery within 48 h upon inclusion.

### Definition of adverse outcomes

The occurrence of any fetal or maternal AOs was the primary outcome. This composite outcome was defined as follows: Maternal AOs comprised HELLP syndrome, disseminated intravascular coagulopathy (DIC), cerebral hemorrhage, pulmonary edema, renal failure, eclampsia, and maternal death. Fetal AOs included respiratory distress syndrome (RDS), necrotizing enterocolitis (NEC), intraventricular hemorrhage (IVH), placental abruption, delivery before 34 weeks of gestation due to preeclampsia and fetal death within seven days after delivery.

### The model

**Fig. 1:**
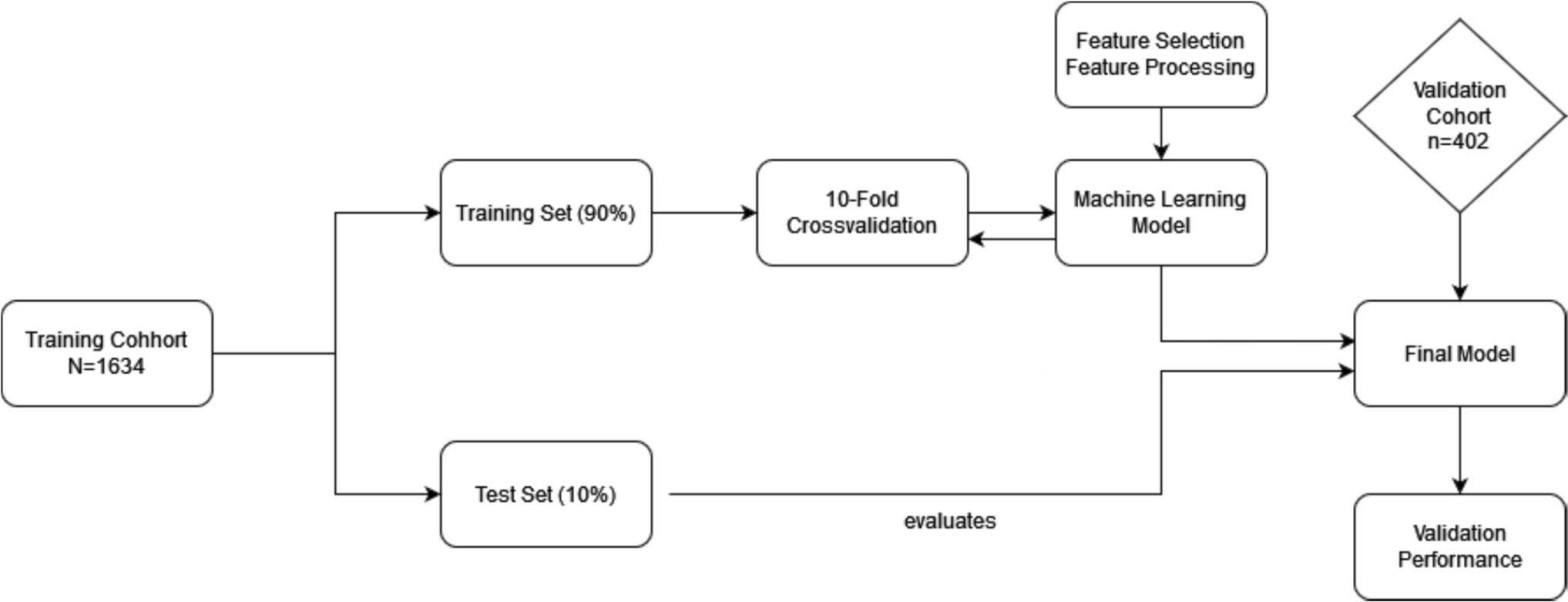
Model development process. The training cohort was split into a training (90%) and test set (10%). Using 10-Fold-Crossvalidation we developed the model while performing feature selection and processing. The attained model was then tested on the remaining 10% of patients. The final model was validated on the validation cohort

This study aimed to adapt the model to predict preeclampsia-related AOs created by Schmidt et al. for clinical use, by reducing the number of outcome features. The model was created using the open-source software library „sklearn.ensemble.GradientBoostingClassifier “ [18]. To train the model, we used a 90%-10% training/test split. Training the model on 90% of the patients and reserving the remaining 10% for a test set. The two groups were sampled randomly, and we took care to only move patient files in their entirety into each separate dataset. We used tenfold-crossvalidation to evaluate the models. Hyperparameter Tuning and Training were done using the open-source Python software library “HyperOpt” [19]. Additionally, logistic regression analysis was employed to train two further models aiming to predict time to delivery: (1) within 14 days in women presenting ≤ 34 weeks, (2) within 7 days in women > 34 weeks. These models were equally trained on a 90%/10% train/test split. Simultaneously, we assessed the most relevant features through ML-methods to train a more feasible model. Figure 1. shows the model development process.

### Feature processing and reduction

The original model by Schmidt et al. comprised of 71 features commonly collected during routine clinical care. It included data from patient history, symptoms at presentation, laboratory results including biomarkers, and ultrasound findings. Additionally, several features were engineered bringing the final feature set to a total of 114 features [13]. To significantly reduce the number of features we calculated Shapley Values for the original and our model to identify relevant features [20, 21]. To this aim, we performed various ML-methods such as recursive feature elimination, feature distribution per target, and feature importances from tree-based method. Mirroring the original model, feature processing was included: We calculated the BMI (kg/m2) and the ratio of sFlt-1/PlGF. Systolic and diastolic blood pressure measurements were expressed as multiples of gestation age-specific median, based on the reference ranges reported here [22]. Similarly, sFlt-1, PlGF and their ratio were included as multiples-of-median of the gestation age [23]. Clinical and statistical experts selected the final list of relevant features. The emphasis for choosing the final features was on high feature performance. Where possible, other factors, such as economic reasons or widespread availability in routine clinical care, were weighed in the decision-making, if it did not result in loss to model performance. The final feature set comprises thirteen raw input features that were partly processed into thirteen variables in the final set. It includes variables on baseline characteristics, laboratory results, gestation age and blood pressure. Table 1 shows the thirteen raw input and their final form.

**Table 1:** List of Raw Input features and the final feature set

Raw input feature	Final feature
Age in years	Age in years
Height in cm	BMI in kg/m^2^
Weight in kg	
Preexisting hypertension Yes / No	Preexisting hypertension Yes / No
New-onset hypertension Yes / No	New-onset hypertension Yes / No
Gestation age in days	Gestation age in days
Urine dipstick in „ + “	Urine dipstick in „ + “
Systolic blood pressure in mmHg	Systolic blood pressure in Multiples-of-Mean
Diastolic blood pressure in mmHg	Diastolic blood pressure in Multiples-of-Mean
sFlt-1 in ng/l	sFlt-1 Multiples-of-Median
PlGF in ng/l	PlGF Multiples-of-Median
	sFlt-1/PlGF Multiples-of-Median
Elevated liver enzymes Yes / No	Elevated liver enzymes Yes / No
Low platelets Yes / No	Low platelets Yes / No

### Missing value imputation

For the training cohort we excluded all cases with more than 50% missing values. On the remaining dataset, we performed missing value imputation. For metric variables, gestation age-specific median values were added and for categorical data missing values were imputed using the mode. No missing value imputation was performed on the validation cohort. After excluding all incomplete cases, it comprised only those cases with no missing values in the 13 raw input features.

### Statistical analysis

We assessed data consistency across training and validation cohort by using the unpaired t-test for continuous data with normal distribution and Mann–Whitney U-test for non-normal distribution. Categorical data was compared using the χ^2^ test. To account for multiple comparisons in the descriptive statistics, Bonferroni correction was applied, and tests were considered significant with a two-sided p-value < 0.05. Effect sizes were calculated for variables with statistically significant differences. The statistical software used was IBM SPSS Statistics (Version 28, IBM, Armonk, USA). We used AUROC, accuracy, F1-Score, sensitivity, specificity, PPV and NPV to judge model performance on the test set and validation cohort. The AUROC for each model was calculated using the equal error rate, balancing the false-positive and false-negative rate. These metrics were acquired using Python and the open-source software library (Version 3.9, Python Software Foundation, Wilmington, USA). Furthermore, we used the DeLong test [24] to compare the AUROC for each model using a two-sided p-value < 0.05. The programming software R and the R package “pROC” [25] was used.

## Results

### Baseline characteristics

The training cohort included 1634 patients with 2412 visits, while the validation cohort included 402 patients and 540 visits.

Overall, both cohorts had a similar distribution across most variables, though gestational age at inclusion (224 and 239 days) and delivery (255 and 263 days) differed significantly (both *p* = 0.008). This is further reflected in the mean gestation age at visit, which was higher in the validation (240 days) than in the training cohort (226 days) (p = 0.008, Cohen’s *d* = 0.174). Furthermore, there was a significant difference in systolic blood pressure between the groups with a mean of 132 in the training cohort and 129 in the validation cohort (*p* = 0.016). According to Cohen, the effect size is considered to be small (*Cohen’s d* of 0.136, and 0.267, respectively) [26]. The difference in urine dipstick measurements (*p* = 0.008) and low platelets (*p* = 0.008) were also significant, while the effect size determined by Cramer’s V (*φ* = 0.198 and *φ* = 0.064, respectively) points to a negligible effect. Table 2 compares baseline characteristics in both cohorts.

**Table 2:** Baseline characteristics evaluated per patient and per visit

	Cohort		
Variable	Training cohort	Validation cohort	*p*
*Per Patient*			
Age in years	32,04 ± 6,08	32,39 ± 5,76	0,304
Weight in kg	73,78 ± 18,61	76,07 ± 19,92	0,424
Height in cm	165,37 ± 6,65	166,15 ± 6,94	1
BMI in kg/m2	26,96 ± 6,56	27,55 ± 6,97	0,968
Gestation age at inclusion	224,15 ± 36,33	239,41 ± 33,15	0,008
Gestation age at delivery	255,20 ± 27,28	263,76 ± 23,20	0,008
Preexisting hypertension	178 (10,9%)	40 (10,0%)	1
New-onset hypertension	481 (29,4%)	142 (35,3%)	0,176
*Per visit:*			
Systolic blood pressure in mmHg	132,00 ± 22,17	129,07 ± 18,71	0,016
Diastolic blood pressure in mmHg	81,81 ± 15,02	77,82 ± 14,74	0,008
sFlt-1	5623,12 ± 5540,00	5169,40 ± 4780,55	1
PlGF	264,26 ± 360,48	272,24 ± 386,60	0,848
sFlt-1/PlGF-ratio	106,06 ± 199,83	73,77 ± 140,39	1
Urine dipstick in „ + “			
Negative	1219 (50,5%)	374 (69,3%)	
Trace	129 (5,3%)	17 (3,1%)	0,008
+	203 (8,4%)	44 (8,1%)	
+ +	164 (6,7%)	54 (10,0%)	
+ + +	201 (8,3%)	201 (8,3%)	
+ + + +	0 (%)	13 (2,4%)	
Low Platelets	128 (5,3%)	50 (9,3%)	0,008
Elevated Liver Enzymes	153 (6,3%)	39 (7,2)	1
Gestation age at visit	225,67 ± 34,35	240,34 ± 31,23	0,008

Continous variables (age, weight, height, BMI and gestation age at inclusion and delivery, blood pressure measurements, sFlt-1, PlGF and their ratio, gestation age at visit) are presented as arithmetic mean ± standard deviation. And were compared using t-test and mann-whitney-u-test (depending on distribution). Categorical data is presented in absolute and relative frequency and was compared using the Chi-squared-test. For variables evaluated per patient n=1634 in the training cohort and *n* = 402 in the validation cohort. For variables evaluated per visit *n* = 2412 in the training cohort and n=540 in the validation cohort.

### Target variables

The distribution of AOs was comparable in both cohorts. In 15.4% of cases in the training cohort we documented a delivery before 34 + 0 weeks due to preeclampsia, compared to 1.0% in the validation cohort (*p* = 0.013, *φ* = 0.173). The training cohort also had a higher rate of “any AO” (24.5%) than the validation one (11.7%) (*p* = 0.013, *φ* = 0.123). Both showed only a small effect size. Table 3 shows the distribution of all AOs between both cohorts.

**Table 3:** Maternal and Fetal Adverse Outcomes

	Cohort		
Adverse outcome	Training cohort *n = *1634	Validation cohort *n* = 402	p
Maternal			
DIC	1 (0,1%)	0 (0%)	1
HELLP	48 (2,9%)	15 (3,7%)	1
Cerebral Hemorrhage	0 (0%)	1 (0,2%)	0,572
Pulmonary Edema	5 (0,3%)	0 (0%)	1
Renal Failure	8 (0,5%)	1 (0,2%)	1
Eclampsia	1 (0,1%)	2 (0,5%)	0,533
Death	1 (0,1%)	0 (0%)	1
Fetal			
RDS	188 (11,5%)	27 (6,7%)	1
NEC	3 (0,2%)	2 (0,5%)	0,065
Placental abruption	12 (0,7%)	4 (1,0%)	1
IVH	5 (0,3%)	3 (0,7%)	1
Premature Birth due to preeclampsia GA < 34 weeks)	251 (15,4%)	4 (1,0%)	0,013
Death	24 (1,5%)	4 (1,0%)	1
Any adverse outcome	401 (24,5%)	47 (11,7%)	0,013

Categorical data is presented in absolute and relative frequency and was compared using the Chi-squared-test. DIC (disseminated intravascular coagulation), HELLP (Hemolysis, Elevated Liver Enzymes, Low Platelets-Syndrome), RDS (respiratory distress syndrome), NEC (necrotizing enterocolitis), IVH (intraventricular hemorrhage), GA (Gestation age).

Of 908 women presenting before 34 weeks 223 were delivered within 14 days (24.6%) for the training cohort compared to the total of 153 in the validation cohort of which 38 (24.8%) delivered in said time frame. The test set contained 147 visits for the first and 202 visits for the validation cohort. In women presenting > 34 weeks of gestation delivery occurred in 353 cases (48.6%) in the training cohort and in 114 (45.8%) in the validation cohort. No significant differences between the two cohorts were found concerning time to delivery.

### Predictive accuracy

**Fig. 2:**
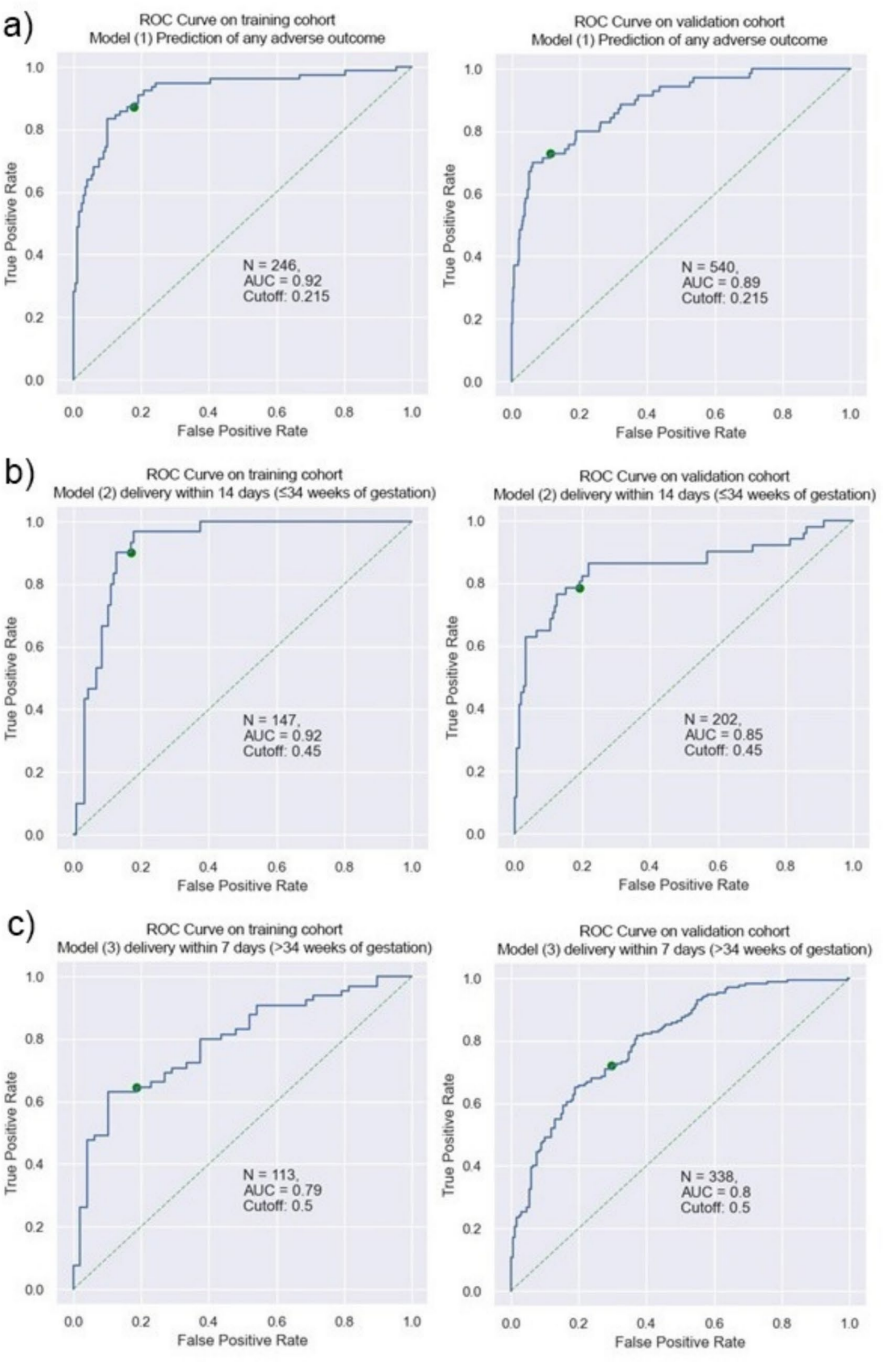
The ROC curves for both the training and validation cohort. **a** Model (1) to predict any adverse outcome was trained on 90% of the visits in the training cohort and was tested on the remaining 10% (246 visits). The AUROC for the training and validation cohort were 0.92 and 0.89 (*p* = 0.31). **b** Model (2) to predict delivery within 14 days prior to 34 weeks gestation was trained on 90% of the visits in the training cohort and was tested on the remaining 10% (147 visits). The AUROC for the training and validation cohort were 0.92 and 0.85 (*p* = 0.13). **c** Model (3) to predict delivery within 7 days from 34 weeks gestation on was trained on 90% of the visits in the training cohort and was tested on the remaining 10% (113 visits). The AUROC for the training and validation cohort were 0.79 and 0.80 (*p* = 0.78)

The model for detecting any AO yielded an AUROC of 0.92 (95% CI; 0.88–0.96) in the training cohort compared to 0.89 (95% CI; 0.84–0.93) in the validation cohort (*p* = 0.31). For delivery within 14 days, the AUROC was 0.92 (95% CI; 0.87–0.96) for the training cohort and 0.85 (95% CI; 0.78–0.92) for the validation cohort (*p* = 0.13). The model performance for delivery within 7 days yielded an AUROC of 0.79 (95% CI; 0.70–0.87) for the training cohort and a 0.80 (95% CI; 0.75–0.85) for the validation cohort (*p* = 0.78). Table 4 shows the performance metrics for all 3 models. Figure 2 visualizes all three model performances as AUROC.

**Table 4:** Performance Metrics for all three models

Target variable	Cohort	AUROC	Accuracy	NPV	PPV	Sensitivity	Specificity	F1
*Any Adverse Outcome*								
	Schmidt et. al (GBTree Model)[13]	0.81	0.87	0.88	0.82	0.68	0.95	0.74
	Training cohort	0.92	0.83	0.93	0.69	0.87	0.82	0.77
	Validation cohort	0.89	0.85	0.96	0.46	0.73	0.87	0.57
*Delivery within 14 days*								
	Training cohort	0.92 (95% CI; 0.87–0.96)	0.84	0.97	0.57	0.90	0.83	0.70
	Validation cohort	0.85 (95% CI; 0.78–0.92)	0.81	0.92	0.60	0.78	0.82	0.68
*Delivery within 7 days*								
	Training cohort	0.79 (95% CI; 0.70–0.87)	0.71	0.62	0.82	0.63	0.81	0.71
	Validation cohort	0,80 (95% CI 0.75–0.85)	0.71	0.72	0.71	0.72	0.70	0.71

## Discussion

### Summary of the main findings

We developed three ML models predicting (1) any AO, (2) delivery within 14 days for women presenting ≤ 34 weeks of gestation and (3) delivery within 7 days after 34 weeks of gestation, using 13 key features. Validation on an additional cohort showed comparable accuracy, despite differences in both cohorts.

### Comparison to existing literature

Multi-marker modelling has proven to be more effective in predicting preeclampsia and related AOs than single parameters or measuring blood pressure and determining proteinuria [12, 27]. It has been applied in both first trimester [28] and second trimester [29] using competing risk models. The algorithm proposed by the Fetal Medicine Foundation (FMF) was developed to predict the occurrence of preeclampsia, rather than associated AOs. Screening by maternal factors alone yielded detection rates of 49% for pre-term preeclampsia and 38% for term-preeclampsia. The detection rate increased substantially by adding uterine artery pulsatility index, mean arterial pressure and PlGF into the model, rising to 75% and 47%, respectively [28]. Although the endpoint differs from our study, this example illustrates how combining clinical and biochemical parameters can enhance predictive performance in hypertensive pregnancy disorders.

Several authors have demonstrated the predictive capabilities of sFlt-1/PlGF for the onset of preeclampsia and its AOs [11, 30–33]. A sFlt-1/PlGF-measurement ≤ 38 has a high NPV of 99.3% for a week and 94.6% for four weeks [11]. The PPV of the sFlt-1/PlGF-ratio at the cut-off of 38 to predict preeclampsia related AOs within four weeks lies at 65.6%. Dröge et al. included the ratio in a multi-marker regression model using a slightly deviated version of our training dataset and were able to reliably predict AOs. The full model yielded an AUC of 0.89 with an NPV of 0.90 and PPV of 0.75. However, complex statistical calculations had to be made to gain a prediction, not lending itself for clinical use [17].

ML enables analysis of large datasets with many variables. The predecessor to our model to predict any AO was described by Schmidt et al. It was based on the same training cohort and comprised 114 features [13]. While our model’s capability to predict AOs improved upon AUROC (0.81 vs.0.89) and NPV (0.88 vs. 0.96), the PPV noticeably decreased (0.82 vs. 0.46), likely due to fewer features, though overfitting would have to be ruled out by further research.

Albeit being a novel approach, ML has been investigated by other researchers, often focusing on the prediction of preeclampsia itself and not AOs. Ansbacher-Feldman et al. studied the use of artificial intelligence and ML methods to predict the risk of developing preeclampsia in first-trimester screening. The AUC for the model of predicting all types of preeclampsia was 0.817 if biomarkers were included [34]. In their study, ultrasound findings, especially pulsatility indices of the uterine arteries, were found to be major predictors of preeclampsia. Sandström et al. likewise employed ML to screen for preeclampsia in a first-trimester setting. Their three models, based on maternal demographic information and medical history, had a moderate success with an AUCs 0.58–0.68 [15].

Other studies have shown the efficacy of ML in predicting preeclampsia and related AOs later in pregnancy [16, 35–38]. A recent study [35] developed prediction models using data collected in the second trimester with target variables being “(1) later-onset preeclampsia: with delivery ≥ 34 weeks; (2) preterm preeclampsia: with delivery < 37 weeks and (3) term preeclampsia: with delivery ≥ 37 weeks”. The XGBoost-algorithm with a full set of features had a performance of AUROC 0.95 (accuracy 0.92, F1-Score 0.571). Moreover, they trained a “questionnaire-based model” including only features from a list of questions that could solely be answered by the patient alone, also investigating the efficacy of a reduced feature set. The model based on these 8 features yielded an AUROC of 0.838 with an accuracy of 0.828 and F1-Score of 0.340 [35]. Montgomery-Csobán et al. [38] used ML to predict AOs for women presenting with preeclampsia within two days, focusing on maternal complications. Their training data included patients from high-, middle-, and low-income countries. Based on 18 features, their model yielded an AUROC of 0.80 (95% CI 0.76–0.84).

Biomarkers have also been studied for time-to-delivery prediction. Rana et al. found that a sFlt-1/PlGF-ratio ≥ 85 occurred in 86.0% of women < 34 weeks' gestation with need of delivery within 14 days [39]. A secondary analysis of the PROGNOSIS cohort also found the sFlt-1/PlGF-ratio at the cut-off of 38 to be an indicator for the remaining pregnancy duration [40]. Villalain et al. also used ML to predict time to delivery. They trained their model not on a high-risk cohort, but on patients with diagnosed early-onset preeclampsia and limited the prediction window to 7 days after diagnosis. Features included data concerning demographic information, biomarkers, and ultrasound findings of pulsatility indices and fetal weight estimate. The study yielded an AUROC of 0.79 compared to 0.85 in our second model. However, they were able to achieve a higher PPV (81.5% vs. 60.0%) [41].

### Strengths and limitations

This study demonstrates the feasibility of using ML to predict AOs in patients with preeclampsia as well as time to delivery. A major strength is using real-world data, employing the algorithm on a dataset that represents its later area of application. We are one of the first to use a clinically meaningful reduced feature set and still yield high results.

While a smaller sample size, retrospective design, and missing value imputation in the training cohort pose limitations, performance remained consistent across cohorts.

Comparing the two datasets, we found some statistically significant differences. We were able to show, however, that these differences were minimal in effect size. Furthermore, performance metrics of the models only deviated moderately between the two cohorts, indicating generalizability beyond the training cohort.

Due to the limited sample size, we opted to perform missing value imputation on the training cohort to increase data for ML. Ideally, datasets would be complete, not necessitating the input of non-real data. We were, however, able to forgo any missing value imputation in the validation cohort, adding to its strength there.

Lastly, our inclusion criteria represent pregnant women at high risk of preeclampsia. We have yet to prove the predictive accuracy of our models on a low-risk population.

## Conclusion

A ML-model using only 13 routinely available features can accurately predict preeclampsia-related AOs and timing of delivery, performing comparably to more complex models. This approach has potential for integration into clinical care to support timely interventions and improve maternal and neonatal outcomes, however, further prospective evaluation is warranted to assess safety, utility and cost-effectiveness.

## Data Availability

Data is available upon request.
